# Optical Probes for Cellular Imaging of G‐quadruplexes: Beyond Fluorescence Intensity Probes

**DOI:** 10.1002/anie.202424931

**Published:** 2025-04-21

**Authors:** Jenna Robinson, Aatikah Majid, Marina K. Kuimova, Ramon Vilar

**Affiliations:** ^1^ Department of Chemistry, Imperial College London Molecular Sciences Research Hub White City Campus 82 Wood Lane London W12 0BZ UK

**Keywords:** Cellular Imaging, Fluorescence Lifetime, Phosphorescence Lifetime, Quadruplex DNA, Ratiometric Probes

## Abstract

The study of G‐quadruplex (G4) structures that form in DNA and RNA is a rapidly growing field, which has evolved from in vitro studies of isolated G4 sequences to genome‐wide detection of G4s in a cellular context. This work has revealed the tangible and significant effects that G4s may have on biological regulation. This minireview describes recent progress in the design of photoluminescent intensity‐independent optical probes for G4s. We discuss the design and use of probes based on fluorescence or phosphorescence lifetime, rather than intensity‐based detection; spectral ratiometric probes; and fluorescent probes for single‐molecule G4‐detection. We argue that each of these modalities improve unbiased G4 detection in cellular experiments, overcoming problems associated with unknown cellular uptake of probes or their organelle concentration. We discuss the improvements offered by these types of probes, as well as limitations and future research directions needed to facilitate more robust research into G4 biology.

## Graphical abstract

DNA and RNA can fold into tetra‐stranded helical structures known as G‐quadruplexes (G4s). There is mounting evidence suggesting that these structures have important biological roles. In this minireview we discuss advances in the design of optical probes for the detection of G4s that offer intensity‐independent readouts that address limitations of conventional fluorescence intensity‐based probes.

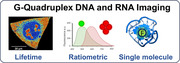

## 1 Introduction

Over the past few years, it has become increasingly evident that DNA and RNA functions are not only dictated by their sequence but also by their secondary structures. For example, while DNA's canonical structure is the archetypal double helix, several other topologies can form during replication and transcription, and play important roles in regulating biological processes.^[^ ^1^ ^]^ In the case of RNA, which displays an even broader range of secondary structures, the specific topology it folds into is essential for defining some of its key properties including interactions with proteins.^[^ ^2^ ^]^

**Figure 1:**
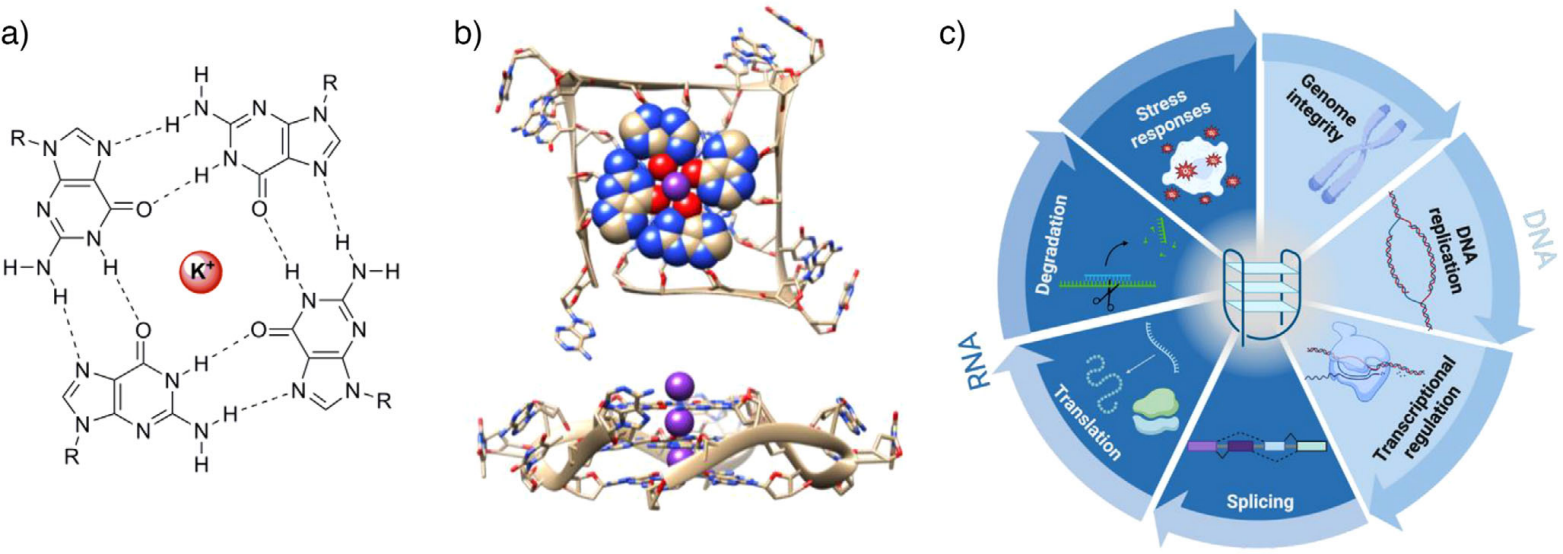
Structure and biological functions of G4s. a) Molecular structure of a guanine‐tetrad highlighting the hydrogen‐bonding interactions and the stabilizing K^+^ cation; b) two views of the X‐ray crystal structure of a G4 DNA structure from the human telomere (structure generated with PyMol; PDB 1KF1); and c) scheme summarizing the biological functions of G4 DNA and RNA. Parts of this figure were created with BioRender.

One of these important non‐canonical secondary structures of DNA and RNA are G‐quadruplexes (G4s), which are tetra‐stranded helical assemblies that come together when four guanine bases hydrogen‐bond with each other by utilizing the Hoogsteen face of each base.^[^ ^3^, ^4^, ^5^ ^]^ G‐quadruplexes are further stabilized by interactions between metal cations, such as K^+^ and Na^+^, and the carbonyl groups of the guanines (see Figure 1a,b). G4s can exist in many forms depending on whether the strands are arranged in a parallel or antiparallel conformation with respect to one another. Additionally, they can form intramolecularly from one strand only, or intermolecularly, when more than one DNA/RNA strands come together via formation of the guanine tetrads.^[^ ^4^ ^]^ The formation of G4s in vitro has been known for several decades.^[^ ^6^ ^]^ Early bioinformatic studies of the human genome revealed approximately 400 000 putative G4‐forming sequences,^[^ ^7^ ^]^ with particular prevalence in promoter regions of genes. Although these studies gave some evidence that G4s might have distinct biological roles, it was the development of G4‐selective antibodies for immunostaining that provided compelling evidence that G4s are present in cells of a broad range of organisms.^[^ ^8^, ^9^, ^10^ ^]^ Subsequently, G4‐selective antibodies have been utilized for immunoprecipitation of G4s from chromatin followed by sequencing (BG4 ChIP‐Seq), which provided genomic resolution of G4 formation.^[^ ^11^ ^]^

The formation of G4s has been implicated in various essential biological processes (see Figure 1c), in animals, plants, bacteria, and viruses. One of the first genomic regions to be associated to quadruplexes in eukaryotes were telomeres,^[^ ^12^, ^13^ ^]^ which are guanine‐rich sequences at the end of chromosomes that act as DNA caps, protecting them from degradation and ensuring their integrity. Telomeric maintenance is a complex process involving enzymes (e.g., telomerase) and capping proteins, and it has been shown that G4 formation in the telomere is important in modulating the function of these proteins.^[^ ^14^, ^15^ ^]^

In addition to telomeres, G4 DNA structures have also been associated with the regulation of gene expression. Bioinformatic studies showed that guanine‐rich regions of the genome with the potential to form G4s are highly prevalent at gene promoter regions and, therefore, it was hypothesized that they could be involved in regulation of transcription.^[^ ^15^ ^]^ Early studies showed that several oncogene promoters contain guanine‐rich sequences that can fold into G4s.^[^ ^16^ ^]^ Stabilizing these G4s with small‐molecule ligands resulted in down‐regulation of the expression of oncogenes,^[^ ^16^, ^17^ ^]^ including *c‐MYC*,^[^ ^18^ ^]^ *KIT*,^[^ ^19^ ^]^ and *KRAS*.^[^ ^20^ ^]^ More recently, it has been shown that the effects caused by stabilization of G4s in promoters is a more complex process than initially proposed. Within cells, global G4 formation is partly controlled by helicase proteins such as BLM, WRN, PIF1, and FANCJ, which actively resolve G4s.^[^ ^21^ ^]^ These enzymes are essential for maintaining G4s at a level that facilitates normal cell function. Excessive stabilization of G4s, for instance by addition of G4‐targeting small molecules or removal of G4 helicases, can in turn lead to DNA damage and general genome instability including chromosomal rearrangements, deletions, and single and double strand breaks.^[^ ^22^, ^23^ ^]^ Therefore, the pharmacological effects observed by molecules targeting G4s in promoters are not only a result of direct regulation of transcription.

G4 DNA‐forming sequences are also highly enriched at origins of replication, being present in 80% of replication sites in humans and mice.^[^ ^24^, ^25^ ^]^ Several studies have shown that for replications to proceed properly, G4s need to be resolved by helicases.^[^ ^26^ ^]^ On the other hand, some studies have suggested that G4s can also be beneficial in replication by interacting with protein complexes which recognize replication origins.^[^ ^25^ ^]^ This highlights the importance of understanding the dynamics of G4 formation/resolution in cells, in order to establish a clear picture of their biological functions.

Although much work was initially focused on G4 DNA, quadruplex‐forming sequences are also prevalent within RNA.^[^ ^27^ ^]^ Although G4s in DNA can form a range of topologies by arrangement of loops in a parallel or antiparallel manner, RNA G4s are almost exclusively present in the parallel form due to the restricted *anti* conformation of glycosidic bonds in ribonucleosides.^[^ ^28^, ^29^ ^]^ RNA G4s have been found to form across the cell in mRNA transcripts and may thus play an important role in RNA translation.^[^ ^30^ ^]^ In addition, G4s have been found to form in essentially all forms of non‐coding RNA including long non‐coding RNAs (lncRNAs),^[^ ^31^ ^]^ such as those associated with telomerase function; in micro RNA (miRNA)^[^ ^32^ ^]^ where G4s can inhibit DICER‐mediated RNA degradation;^[^ ^33^ ^]^ ribosomal RNA (rRNA) which is notoriously guanine‐rich.^[^ ^34^ ^]^ In introns and exons, G4s can also alter splicing isoforms, acting as splicing activators or repressors depending on the genomic context.^[^ ^35^, ^36^, ^37^ ^]^ Finally, emerging research has also shown that G4 RNA play a role in the cellular response to stress.^[^ ^38^ ^]^

**Figure 2:**
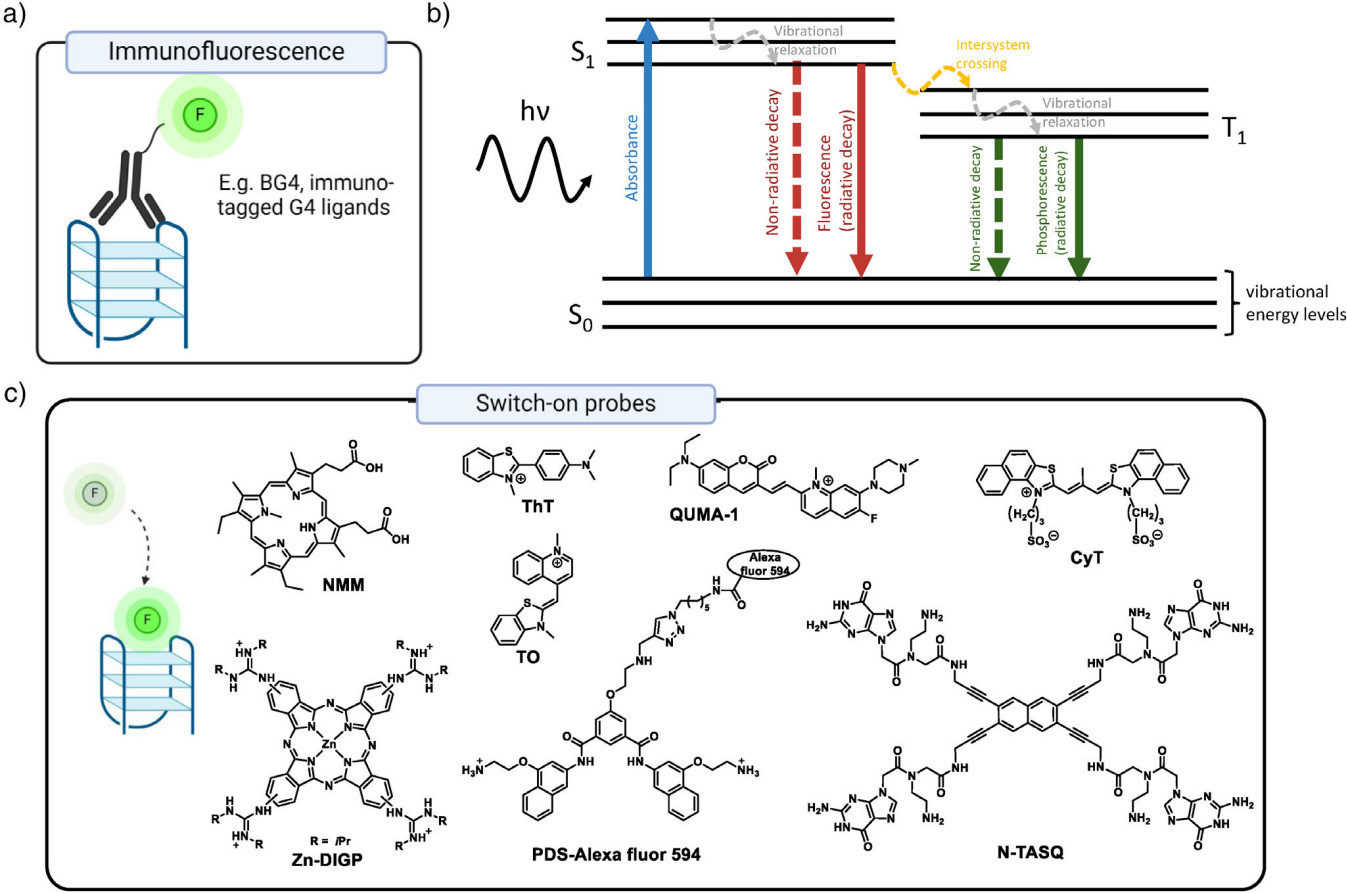
Intensity‐based G4 optical probes. a) A schematic of immunofluorescence‐based approaches, whereby G4s are being targeted by specific antibodies tagged with fluorescent markers; b)–c) Small molecule switch‐on probes, in which fluorescence or phosphorescence emission is enhanced upon G4 binding, due to the suppression of the nonradiative decay; possible decay pathways are shown in a simplified Jablonski diagram in b); examples of various classes of switch‐on probes are given in c). Parts of this figure were created with BioRender.

The implication of G4s in this wide range of biological functions,^[^ ^5^, ^23^, ^39^ ^]^ has stimulated the development of increasingly sophisticated G4‐detection techniques to gain better insights into the biological roles of G4s.^[^ ^40^ ^]^ Microscopy‐based techniques have been developed to globally visualize G4s in cells.^[^ ^40^, ^41^, ^42^ ^]^ Primarily, these microscopy methods utilize i) immunofluorescence with G4‐binding antibodies or ii) small‐molecule fluorescent probes in which direct binding of the probe to G4s triggers a change in its photophysical properties (Figure 2). The most common approach for G4 immunofluorescence visualization utilizes the BG4 antibody.^[^ ^9^ ^]^ In these experiments, cells are first fixed with formaldehyde, permeabilized with detergent and then incubated with BG4 that contains a small peptide “flag‐tag”. Incubation with secondary antibodies is then carried out, which recognize this flag‐tag; additionally the secondary antibodies contain a fluorophore which thus images BG4 binding sites.^[^ ^9^ ^]^ This approach can also be adapted for detection of G4 RNA by treating the cells with DNase and thus, redirecting BG4 binding to the cytoplasm.^[^ ^43^ ^]^ Alternatively, G4 ligands may be used to achieve immunofluorescence, by conjugation of ligands with a BrdU immunotag which is selectively recognized by antiBrdU antibodies.^[^ ^44^ ^]^

However, one of the limitations of antibody‐based mapping strategies is the need for cell fixation and membrane permeabilization, which prevent probing of G4 dynamics within live cells. In contrast, small‐molecule fluorescent or phosphorescent probes can be designed and optimized to cross the cell membrane without any permeabilization steps and therefore enable imaging G4s in their “native” environment.^[^ ^41^, ^42^, ^45^ ^]^ The most common form of small‐molecule G4 probes are so called “switch‐on” molecules, which exhibit a significant enhancement of their emission intensity upon G4 binding (Figure 2c).^[^ ^41^, ^42^, ^45^, ^46^ ^]^ For these probes, binding to G4s affects the equilibrium between the radiative decay, where excited molecules relax to the ground state via a release of a photon, and the non‐radiative decay – where excited energy is released via fast rotation about flexible bonds, or heat‐exchange with the environment. For many of these probes, in an aqueous environment the rate of the nonradiative decay is fast (e.g., via coupling to vibrational modes of water, or FRET with water)^[^ ^47^ ^]^ and no fluorescence is observed. However, if non‐radiative decay is impaired, for example via rotational restriction upon G4 binding, or via protecting the probe from quenching by water molecules inside DNA, the probe exhibits a significant increase in fluorescence intensity.^[^ ^48^ ^]^

G4 fluorescent switch‐on probes generally include large planar, conjugated systems that allow for selective stacking on‐top of G‐quartets^[^ ^41^ ^]^ and have been developed based on diverse fluorescent cores such as thiazole orange (**TO**),^[^ ^49^, ^50^, ^51^ ^]^ thioflavin T (**ThT**),^[^ ^52^ ^]^ N‐mesoporphyrin IX (**NMM**),^[^ ^53^ ^]^ and various metal complexes, in which emission is quenched by water in the absence of the G4 binding.^[^ ^41^, ^45^, ^54^ ^]^ For G4 RNA, additional probes have been developed, such as **N‐TASQ** – whose structure is a biomimetic of a G‐quartet,^[^ ^27^, ^55^ ^]^ as well as **QUMA‐1** ^[^ ^56^ ^]^ and **CyT**.^[^ ^57^ ^]^ More recently, the attempts at detection of G4s in particular organelles, such as mitochondria, have been made by conjugation of G4 small molecule probes with organelle‐targeting tags.^[^ ^58^ ^]^ Fluorescent probes that may be used in tissues and whole organisms have also been described.^[^ ^58^, ^59^, ^60^ ^]^

Another broad class of fluorescence intensity‐based probes comprises conjugates where a nonemissive G4 binder is linked in situ to a fluorescent probe, which is not involved in the direct interaction with the targeted nucleic acid. The first example of this approach was a probe based on linking a derivative of pyridostatin (a well‐known G4 DNA binder) with a fluorescent probe Alexa Fluor 594 via in situ click chemistry.^[^ ^61^ ^]^ This seminal work was followed by several examples where other G4 binders were derivatized with alkynes or azides to subsequently conjugate them to fluorescent probes in cells (via in situ click reactions).^[^ ^62^, ^63^, ^64^ ^]^ This approach has provided important information of the cellular localization and G4 interactions of the molecules under study.

Although intensity‐based probes have been instrumental to study G4s in vitro, their use to reliably detect G4s within cells is more challenging. The first problem is one of selectivity, since most small molecule‐based G4 probes display some degree of binding with duplex DNA which can also lead to fluorescence switch on, even if this is larger when bound to G4s. Given that G4s exist as relatively rare entities in a dense and structurally diverse cellular environment, exceptionally high binding affinity and selectivity for G4s versus duplex is required to detect them in cells. By considering the abundance of G4s relative to other cellular structures, it has been estimated that a 1000‐fold selectivity for G4s over duplex DNA is required for adequate imaging within cells.^[^ ^45^ ^]^ Although such G4 binding selectivity may be obtained with antibodies,^[^ ^9^ ^]^ currently, almost no small‐molecule fluorescent probes meet this criterion. This means that the fluorescent signals measured when using “switch‐on” molecules in cells are likely due, at least in part, to their interactions with duplex DNA or other structures that are considerably more abundant compared to G4s.

To achieve the specificity required for robust cellular imaging, strong G4 binders are needed, with binding affinities in the sub micro‐molar range.^[^ ^45^ ^]^ However, creating G4 probes with such high affinity is not only challenging, but poses the issue of potentially artificially inducing G4 formation. As small‐molecule fluorescent probes are often used in live cells, those that display particularly high binding affinity may in fact perturb the natural homeostasis of G4 formation within cells, putting into question their use for studying the roles of endogenous G4s. For instance, multiple studies have shown that pre‐treatment of cells with strong G4 binders increases the number of BG4 foci,^[^ ^9^, ^43^ ^]^ providing evidence that small molecules that bind to G4s can trigger unnatural G4 formation.

Furthermore, cellular fluorescence intensity measurements are not solely dependent on the degree of target binding, but also on probe uptake into cells and organelles.^[^ ^65^ ^]^ In this regard, the source of an increase in fluorescence intensity is not unambiguous and may be due to either a probe binding to G4s or an increase in local probe concentration. This point is particularly important as more recently G4s have been implicated in triggering liquid–liquid phase separation events within cells.^[^ ^66^, ^67^, ^68^ ^]^ However, phase‐separated organelles may also sequester small‐molecules at high concentration, which makes detection of fluorescent changes due specifically to G4 formation even more challenging.

To address some of the limitations of fluorescence intensity‐based optical probes, alternative approaches of G4 imaging have been developed. This includes the use of fluorophores based on fluorescence lifetime (rather than intensity); spectral ratiometric probes; and single molecule‐based G4‐detection methods, which each help to address the limitations associated with the use of fluorescence intensity switch‐on probes highlighted above. The scope of this review is to discuss advances in these three approaches for the visualization and quantification of G4 structures in cells. For comprehensive coverage of the literature on fluorescence intensity‐based probes we refer the reader to several previous reviews.^[^ ^41^, ^42^, ^45^, ^46^ ^]^

## 2 Fluorescence Lifetime Probes

**Figure 3:**
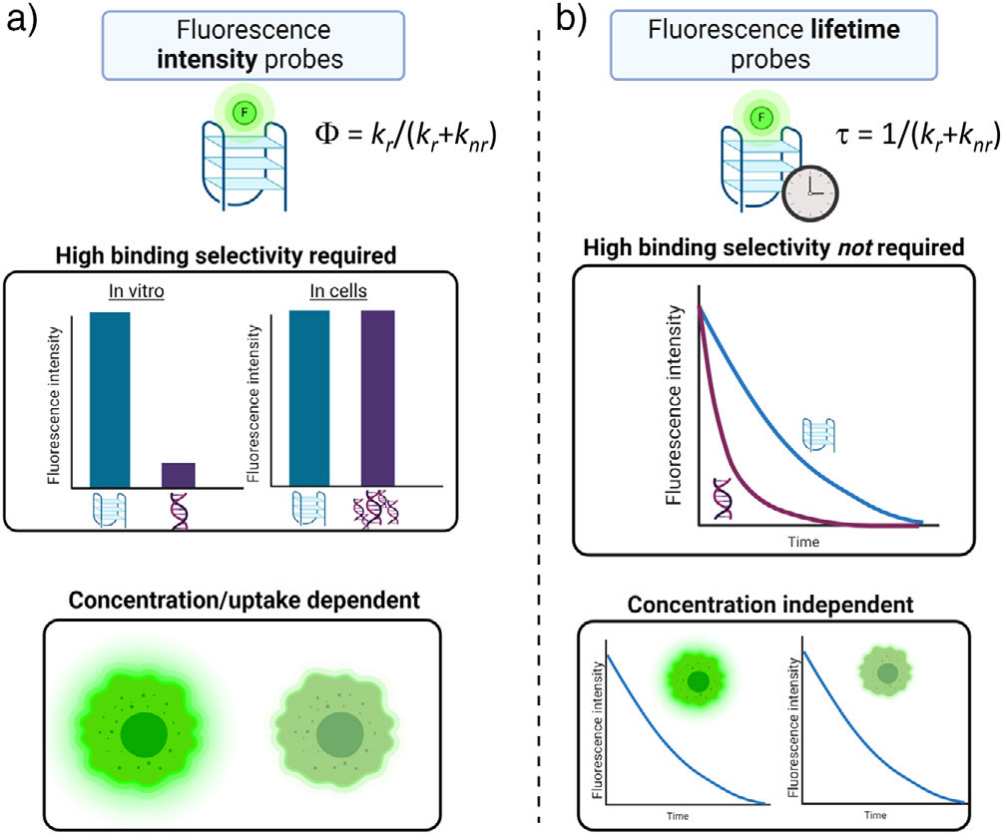
A comparison of fluorescence intensity‐based a) and lifetime‐based b) G4 probes. The signal of intensity‐based probes is based on quantum yield, Φ, where k_r_ and k_nr_ correspond to the radiative and nonradiative rate constants, respectively and is concentration dependent. In contrast, lifetime‐based probes (detecting lifetime, *τ*) allow concentration‐independent measurement. This makes this method particularly suitable for cellular detection, where the cellular and organelle uptake of the probe can vary. Created in BioRender.

Fluorescence lifetime imaging microscopy (FLIM) has emerged as a new approach for cellular visualization of G4s, which capitalizes on the fact that G4 binding can induce changes to the rate of fluorescence decay of a probe, as well as its fluorescence intensity (Figure 3). Fluorescence lifetime (τ) refers to the average time a fluorescent molecule spends in the excited state.^[^ ^48^, ^69^ ^]^ The fluorescence decay as a function of time (t) can be measured following pulsed excitation, which is then fitted to a (multi)exponential decay curve (Equation 1), with amplitudes α_i_ and lifetime components (τ_i_) as parameters. The fluorescence lifetime of a molecule is determined by the rates of radiative and nonradiative decay (Figure 3) similarly to fluorescence intensity.

(1) $$F\left(x\right)=\Sigma \alpha_{i}e^{\text{-(t/}\;\tau \;\text{i)}}$$

Provided the fluorescence lifetime of a probe interacting with G4 structures is distinctly different to that induced by non‐G4 interactions, a probe's lifetime can be used to identify G4 binding. Typically, G4 binding results in a significant reduction of the nonradiative decay of such probes, and the resultant lifetime is significantly longer than in any other scenario (e.g., upon changes in polarity, viscosity, or binding to other macromolecules). This allows for the unique identification of G4s, even in the presence of many competing interactions. Therefore, fluorescence lifetime‐based probes do not need to bind exclusively to G4s, but simply to exhibit a unique lifetime signature when bound to G4s that allows such interactions to be distinguished. In turn, the requirement of high binding affinity and selectivity is not essential when using fluorescence lifetime‐based probes. In addition to this, the fluorescence lifetime of a molecule is generally independent of the probe's concentration as the fluorescence decay rate is an intrinsically ratiometric parameter.^[^ ^48^ ^]^ Therefore, fluorescence lifetime measurements eliminate the concern that differences of probe uptake into cells may confound measurements of G4 abundance. Overall, this makes fluorescence‐lifetime based probes robust tools for G4 detection which are independent of a probe's uptake and of its binding selectivity to G4s versus other structures (Figure 3).

**Figure 4:**
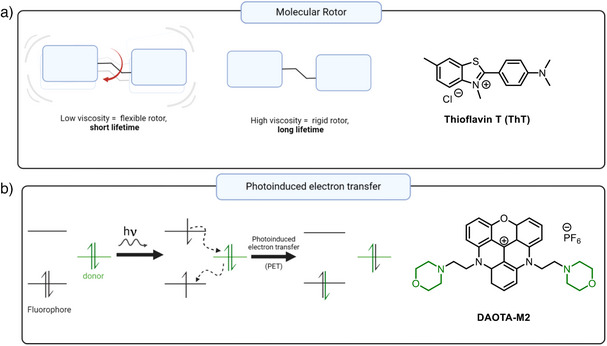
The basic principles of molecular rotor type sensing a) versus photoinduced electron transfer (PET) type sensing b) of G4s. Molecular rotor‐based probes respond to rigidification of their structure due to G4 binding by increased lifetimes; **ThT** is a representative example of this class of probes.^[^ ^76^ ^]^ PET‐based probes are nonemissive prior to binding due to an efficient PET between its constituent parts, while binding to G4 separates the donor and acceptor moieties in space, leading to increased lifetime. **DAOTA‐M2** ^[^ ^78^, ^79^ ^]^ is a representative example of this class of probes. Created in BioRender.

The fluorescence lifetime of a probe may be influenced by multiple environmental factors.^[^ ^70^ ^]^ For instance, in some fluorophores (termed molecular rotors), fast non‐radiative decay dominates when conformationally‐flexible fluorophores are free in solution (Figure 4a).^[^ ^71^ ^]^ This high rate of non‐radiative decay results in very short fluorescence lifetimes, as well as low fluorescence intensities. However, when intramolecular rotation is inhibited, for instance when conformational freedom is restricted upon binding to biomolecules such as DNA, the fluorescence lifetime and fluorescence intensity increase dramatically. A number of lifetime‐based probes that utilize this principle have been recently reported,^[^ ^72^, ^73^, ^74^, ^75^ ^]^ including the molecular rotor **ThT**,^[^ ^76^ ^]^ (shown in Figure 4a) and **TOR‐G4** (see Figure 7a).^[^ ^77^ ^]^ Importantly, probes of this type are selected such that the effect G4 binding has on intramolecular rotation far exceeds the effect of viscosity within cellular organelles, which allows for specific G4 detection.

In addition to internal rotation, emission lifetimes can be reduced by various excited state quenching processes.^[^ ^48^, ^69^ ^]^ For example, photoinduced electron transfer (PET) is known to be strongly conformation‐dependent. During PET, an electron from the higher lying molecular orbital relocalizes to an acceptor group, resulting in excited state quenching (Figure 4b). Changes in the binding conformation of a probe can trigger changes in PET efficiency and, in turn, result in significant alterations to the probe's fluorescence lifetime. For example, the **DAOTA‐M2** family of lifetime‐based G4 probes (see below) is based on this principle and we were able to demonstrate that in some of these dyes the conformation change upon G4 binding drives a large lifetime change that allows G4 detection in live cells.^[^ ^78^, ^79^, ^80^ ^]^

Like with any G4 probe, it is essential to ensure that the effect of G4 binding has on the photophysics of the FLIM‐based probes, dominates over effects from any other environmental factors, such as polarity, viscosity or pH.^[^ ^71^ ^]^ For example, in the case of **DAOTA‐M2**, the pH‐dependent protonation of morpholino group eliminates PET quenching and results in long fluorescence lifetime. However, it could be shown that pH has no effect on G4‐bound probe.^[^ ^80^ ^]^

**Figure 5:**
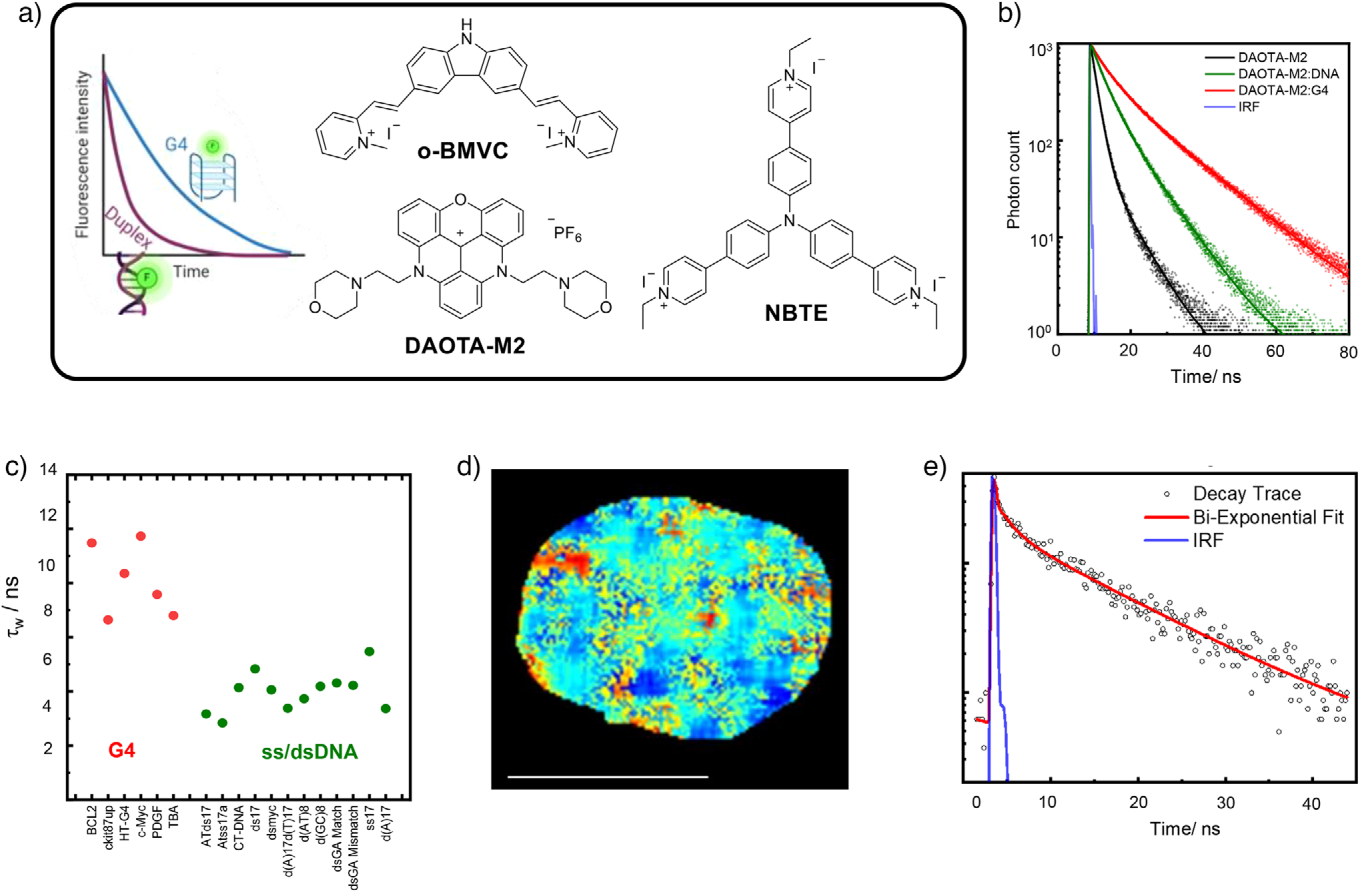
Working principles of lifetime‐based G4 probes. a) The cartoon depiction of the lifetime increase of lifetime probes such as **o‐BMVC**, **DAOTA‐M2,** and **NBTE** ^[^ ^72^, ^74^, ^79^ ^]^ upon G4 binding; b) time‐resolved fluorescence decay traces recorded for **DAOTA‐M2** in aqueous buffered solution (black), compared to in the presence of duplex CT DNA (green) and *c‐myc* G4 DNA (red); c) the distribution of average lifetimes recorded for **DAOTA‐M2** in the presence of six G4 topologies and twelve nonG4 DNA (duplex, single stranded), with all G4s inducing significantly longer lifetimes of the probe, which is sufficient for cellular G4 differentiation via fluorescence lifetime imaging microscopy (FLIM); d) a typical FLIM image of the nucleus of a single live U2OS cell stained with **DAOTA‐M2**, the colour scale is from 9 (red) to 13 ns (blue) (scale bar 20 µm); e) a typical time resolved decay trace recorded from the nucleus of a cell, with biexponential fit, indicating the presence of G4 DNA in the nucleus. Copyright Nature PG 2021. Panels A) and F) created in BioRender. Data reproduced with permission from Summers et al.^[^ ^78^ ^]^

Although there are many benefits of using fluorescence lifetime probes compared to intensity‐based switch‐on probes, currently there are only a limited number of G4‐specific probes which use this approach,^[^ ^72^, ^73^, ^74^, ^75^, ^77^, ^79^, ^81^ ^]^ in stark contrast to the hundreds of fluorescence intensity‐based probes that have been reported.^[^ ^41^, ^42^, ^45^ ^]^ The first published example of a G4 fluorescence lifetime probe was **o‐BMVC** (Figure 5a)^[^ ^73^, ^74^, ^82^ ^]^ whose fluorescence lifetime more than doubled in the presence of G4 structures (∼2‐4 ns) compared with duplex DNA (∼1 ns). However, the probe did not show uptake into live cells and limited nuclear staining was observed within fixed cells, with mitochondrial localization of the probe visible. Despite the modest nuclear staining, the probe was later used to study DNA G4 formation within fixed cells of human‐derived cell lines and in patient‐derived tissue samples where they found significantly higher G4 formation in cancer samples.^[^ ^82^ ^]^

In 2015 a new G4 fluorescence lifetime probe, **DAOTA‐M2**, was published, which as mentioned above was based on a triangulenium fluorescent core and operated based on PET quenching between the core and peripheral morpholino groups (Figure 4b).^[^ ^78^, ^79^, ^80^, ^83^ ^]^ When free in solution, the triangulenium core in **DAOTA‐M2** undergoes an efficient PET quenching with the free‐moving morpholino substituents, resulting in low quantum yield and short lifetimes. However, upon binding to DNA, the morpholino substituents are “fixed” in space at the largest possible distance from the emissive core, preventing PET with the core, which causes a large switch‐on effect in the fluorescence intensity and a significant lengthening of the probe's fluorescence lifetime. Interestingly, the non‐covalent interactions displayed by **DAOTA‐M2**’s morpholino groups with duplex and G4 DNA are not the same, leading to clear differences in the fluorescence lifetime when bound to each of these topologies. These interactions result in **DAOTA‐M2**’s lifetime being in excess of 11 ns in the case of G4 binding, compared to 5–7 ns in the case of duplex DNA binding and 1 ns for **DAOTA‐M2** free in aqueous buffered solution (Figure 5b,c). These very large differences of lifetime seen between different topologies (Figure 5c), allowed for a clear identification of G4 structures even in the presence of a 100‐fold excess of duplex DNA in vitro,^[^ ^78^ ^]^ providing a way for detecting G4 DNA in live cells (Figure 5d,e).

**Figure 6:**
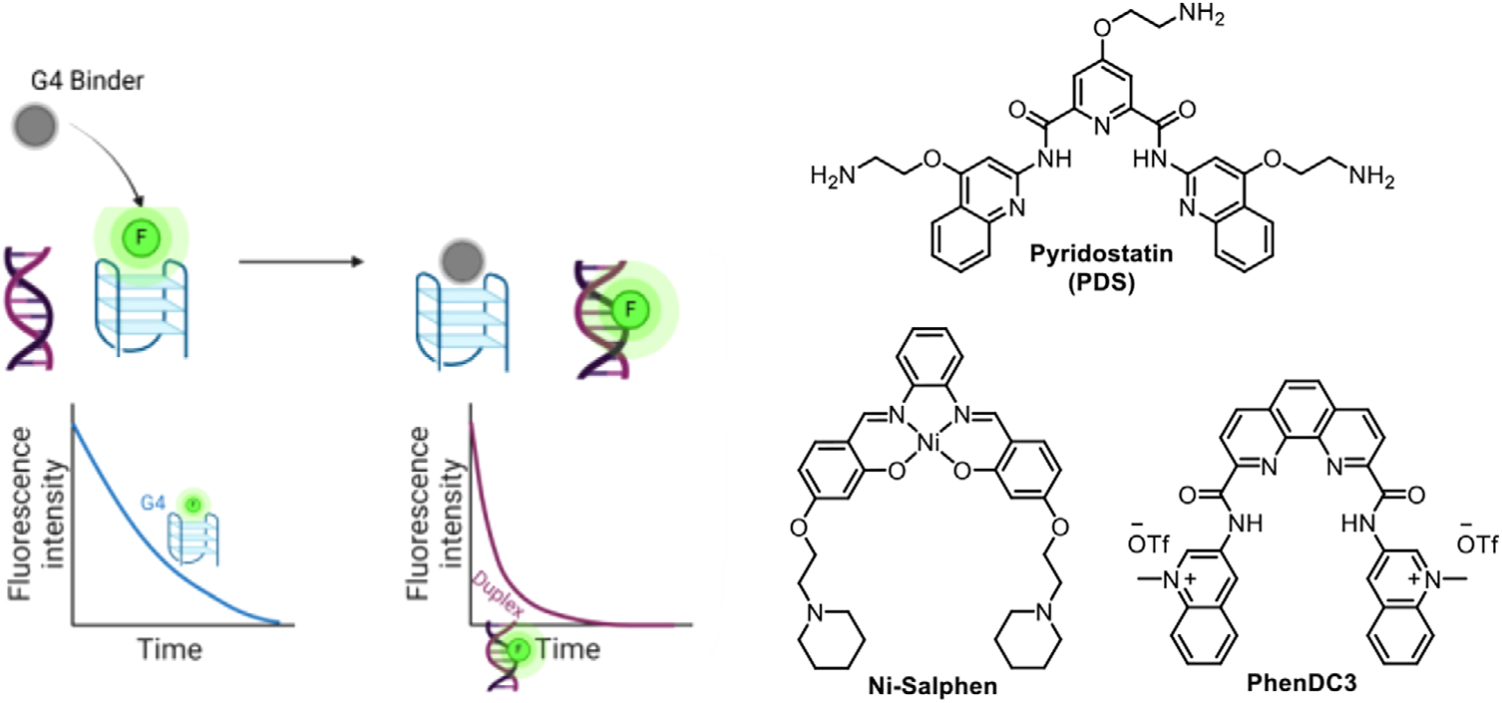
A cartoon representation of the working principles of a fluorescence lifetime indicator displacement assay (FLIDA), whereby a strong G4 binder can displace a fluorescence lifetime probe (e.g., **DAOTA‐M2**) from G4, causing a reduction in lifetime that can be used to screen the binding strength of nonfluorescent G4 ligands; the structures of strong binders **PDS**, **Ni‐Salphen**, **and PhenDC3** complexes are shown.

Validation of **DAOTA‐M2** as a G4‐specific probe was achieved by displacing the probe from G4s using strong known G4 binders, such as pyridostatin (**PDS**)^[^ ^84^ ^]^ and **Ni‐Salphen** (Figure 6).^[^ ^85^, ^86^ ^]^ Increasing amounts of these competitive binders resulted in correspondingly larger reductions in the fluorescence lifetime of the probe due to its displacement from G4 DNA, measured in vitro and *in cellulo*. The reduction in lifetime upon addition of **PDS** and **Ni‐Salphen** indicates that fewer G4s are available for **DAOTA‐M2** binding after the treatment and, hence, this probe, unlike G4‐specific antibodies, competes for the same G4 binding sites as **PDS** and **Ni‐Salphen**. On the contrary, pretreatment of cells with **PDS** was shown to increase the number of **BG4** and **o‐BMVC** foci.^[^ ^73^, ^74^, ^82^ ^]^ Additionally, **DAOTA‐M2** showed very good cellular uptake in live cells and uniform nuclear staining, which was not impacted by cellular treatment with RNase,^[^ ^78^ ^]^ thus making this probe ideal for investigating DNA G4 formation in cells.

Furthermore, the lifetime‐sensitivity of **DAOTA‐M2** to G4s, was demonstrated by the knockdown of G4‐helicases RTEL1 and FANCJ which resulted in significant increases in cellular G4 formation, with double knockdown resulting in the most significant increase of G4 levels in cells (measured by longer lifetimes of **DAOTA‐M2**).^[^ ^78^ ^]^

We note that as with any biophysical approaches, it is important to analyze all FLIM data for statistical significance. Thus, the number of independent repeats, as well as the quality of the data will determine the detection limit. Although knockdown of helicases only induced a small increase in lifetime (0.3–1 ns), the increase was statistically significant and the lifetime increase in each case correlated with the lack of helicase expression in the cells tested.^[^ ^78^ ^]^

Another conformationally flexible fluorescence lifetime‐based probe for cellular imaging of G4s is a tripodal cationic dye **NBTE** (Figure 5A), which also exhibits selectively high lifetimes when bound to G4s compared to duplex DNA.^[^ ^72^ ^]^ Molecular modelling studies demonstrated that the probe interacts with duplex DNA via groove binding, which engaged just two of the three arms of the molecule. In contrast, binding to G4s by end‐stacking on top of G‐quartets, involved all three arms of the probe. The G4 binding interaction thus induced larger rotational constraints on the molecule and lengthened the probe's fluorescence lifetime. The lifetime distinctions exhibited by **NBTE** when interacting with different nucleic acid structures could therefore be used to image G4s in human lung, breast, and liver cells where they also found significant elevation of G4s in cancer cell lines relative to the healthy control cells.^[^ ^72^ ^]^

Recently, it was demonstrated that time‐resolved decays of commercially available probes thiazole orange (**TO**) and thioflavin T (**ThT**) are distinctly different depending on whether they bind to duplex or G4 DNA and RNA.^[^ ^81^ ^]^ Although this makes them potential probes for FLIM‐based G4 detection in cells, only **ThT** was shown to work as a G4 probe in live and fixed cultured cells, due to a significant aggregation of **TO**, which prevented reproducible lifetimes to be measured. Large variations in cellular lifetimes were observed upon treatment with RNase and DNase (in fixed cells) and upon competitive displacement of the probe with **Ni‐Salphen**, confirming **ThT’**s binding to G4s.

As mentioned in the introduction, G4s also form abundantly within RNA (rG4s) and their formation has been linked to the regulation of RNA splicing, translation and export as well as RNA‐mediated cellular stress responses.^[^ ^5^, ^27^, ^87^ ^]^ However, due to the increased structural diversity of RNA relative to DNA^[^ ^2^ ^]^ there is an even greater risk of false positive signals when using traditional switch‐on optical probes to detect rG4s.

**Figure 7:**
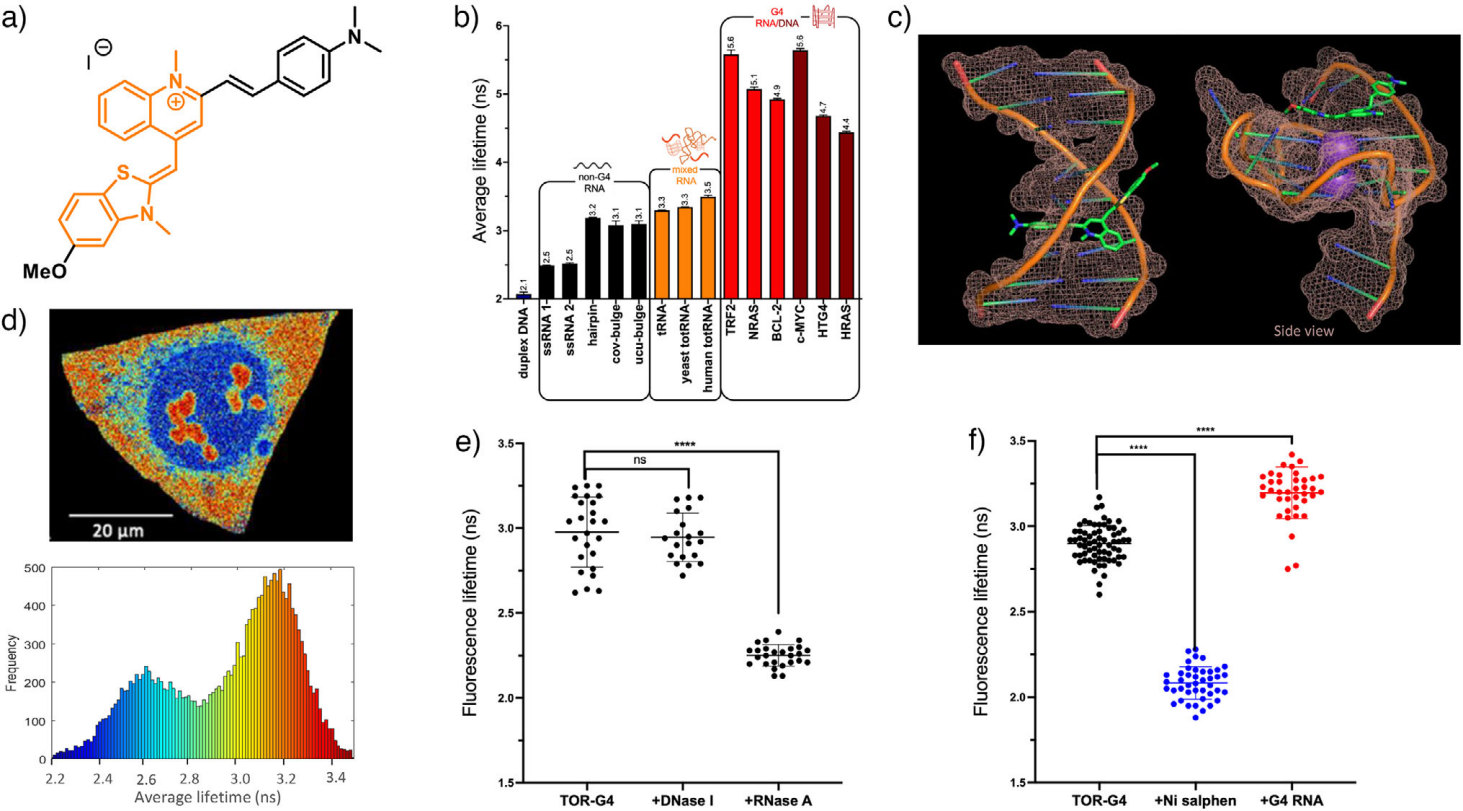
Detecting G4 RNA with TOR‐G4.^[^ ^77^ ^]^ a) The molecular structure of **TOR‐G4**, with the thiazole orange (TO) core shown in orange; b) the distribution of average lifetimes recorded for **TOR‐G4** in the presence of duplex DNA and various RNA topologies, G4s show significantly longer lifetimes sufficient for cellular G4 differentiation via fluorescence lifetime imaging microscopy (FLIM); c) molecular modelling of **TOR‐G4** bound to duplex DNA (left) and G4 RNA (right), showing the probe adopts different conformations, which explains distinct lifetimes seen upon G4 DNA/RNA binding; d) a typical FLIM image of a single fixed U2OS cell stained with **TOR‐G4**, the color scale is from 2.2 (blue) to 3.5 (red) ns, shown as a lifetime histogram (bottom), the longest lifetimes are seen in the cytoplasm and nucleoli, consistent with RNA G4 binding *in cellulo*; e) FLIM data from **TOR‐G4** in fixed U2OS cells in the presence of DNase I (no change) and RNase A (large lifetime reduction), confirming that the dye stains cellular RNA, and f) FLIM data from **TOR‐G4** in fixed U2OS cells in the presence of **Ni‐Salphen** (strong G4 binder, resulting in displacement of **TOR‐G4** from cellular G4s, leading to lower lifetime) and after transfection with additional G4 RNA (showing an increase in cellular lifetime consistent with additional RNA G4s detected). Data reproduced with permission from Ref. [77].Copyright American Chemical Society 2024.

Recently, the conformationally‐flexible molecular rotor **TOR‐G4** (Figure 7a) was developed as the first RNA‐selective G4 fluorescence lifetime probe.^[^ ^77^ ^]^ When bound to G4s (both DNA and RNA), **TOR‐G4** displayed a significantly higher fluorescence lifetime (∼5 ns) compared to the lifetime in the presence of nonG4 RNA (∼2–3 ns) or duplex CT‐DNA (∼2 ns, Figure 7b). These distinct fluorescence lifetimes likely arise from differences in the binding conformation of this probe when interacting with G4s compared to nonG4 structures (as modelled via molecular docking simulations, Figure 7c), which, in turn, result in changes to the rate of nonradiative decay. Interestingly, **TOR‐G4** was observed to accumulate in RNA‐rich regions of the cell including the cytoplasm and nucleoli (Figure 7d) and colocalized with commercially available RNA stains. Treatment with RNase (but not DNase) and inhibition of RNA transcription resulted in large changes in the fluorescence intensity and lifetime of the probe, demonstrating its RNA specificity in cells (Figure 7e). As is the case for the DNA G4 probe **DAOTA‐M2**, an addition of strong competitive binders (i.e., **Ni‐Salphen** and **PhenDC3** – see Figure 6) resulted in a significant drop in fluorescence lifetime, which can be attributed to the displacement of **TOR‐G4** from G4s (Figure 7f). Conversely, increasing G4 concentration by transfection of G4 RNA into cells resulted in higher fluorescence lifetimes of the probe measured by FLIM (Figure 7f). Together these results demonstrated that **TOR‐G4** is a useful molecule for concentration‐independent probing of RNA G4s, however, the high toxicity of the probe makes its use limited to imaging fixed cells and tissues.

A cytoplasmic G4 fluorescence lifetime probe has also been developed based on the **NBTE** core, described above for measuring G4 DNA presence. By addition of terminal phosphorus groups to the tripodal core, the localization of the new probe (**TTPP**) was restricted to the cytoplasm.^[^ ^75^ ^]^ However, unlike **TOR‐G4**, the fluorescence of **TTPP** was sensitive to both DNase and RNase treatment, suggesting that the probe may additionally target non‐nuclear DNA structures such as those found in mitochondria. It was found that the fluorescence decay of **TTPP** should be fitted to a biexponential decay model and the second lifetime component (*τ*_2_) of **TTPP** could be used to distinguish probe interactions with G4s (lifetime = 4–5 ns) compared to non‐G4s (lifetime = 3–4 ns). Furthermore, this lifetime component could be reduced by addition of G4 binder PDS or by inducing apoptosis via cisplatin treatment, which resulted in an increase in probe lifetime.

The commercially available probe **ThT** also responded to both DNase and RNase treatment of fixed cells, by alterations in lifetime.^[^ ^81^ ^]^ A large decrease in nuclear lifetime (as well as disappearance of nucleolar staining) was detected after the RNase treatment, while DNase treatment increased nuclear lifetime (the chromatin/nucleoli staining remained unchanged).This data indicates that both DNA and RNA quadruplexes are being stained by **ThT**, with the majority of RNA G4 signal coming from nucleoli.

## 3 Phosphorescence Lifetime Probes

**Figure 8:**
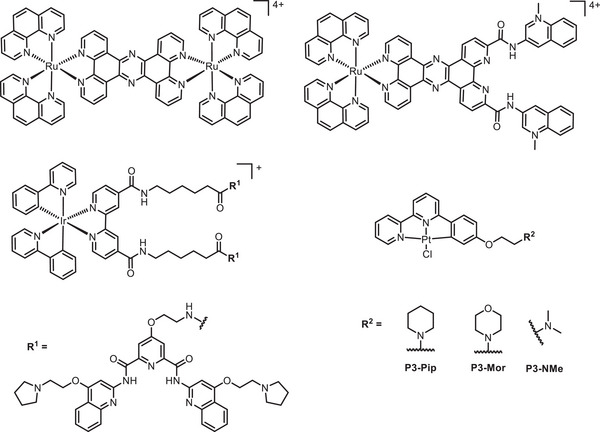
Chemical structures of phosphorescence lifetime metal complexes that have been used to study G4s.

Although fluorescence is the most common form of radiative decay for small organic fluorophores, some molecules, particularly those that contain heavy atoms, can exhibit phosphorescence (Figure 2b).^[^ ^48^, ^88^ ^]^ The latter arises due to spin‐forbidden relaxation of a molecule's excited state that occurs across longer times scales, with characteristic decay constants of up to milliseconds, rather than the nanoseconds typical for fluorescence. Given distinctive timescales of long‐lived phosphorescence, detecting this type of decay gives certain advantages, such as measuring lifetimes that are not contaminated by cellular autofluorescence, and the ability to measure probe's lifetimes alongside other fluorescent markers, even when spectral overlap is present. It has been demonstrated that the phosphorescence lifetime of a molecule may vary upon G4 binding, and the change in lifetime can be detected in a spatially‐resolved manner using phosphorescence lifetime imaging microscopy (PLIM). PLIM‐based G4 probes, based on ruthenium(II),^[^ ^89^, ^90^ ^]^ iridium(III)^[^ ^91^, ^92^ ^]^ and, more recently, platinum(II) complexes^[^ ^93^ ^]^ have been developed to study G4s (Figure 8). The first example of a phosphorescence‐lifetime probe for G4s was based on a di‐nuclear Ru^II^ complexes which displayed a significantly longer phosphorescence lifetime when bound to G4 DNA (*τ* = 129 ns) as compared to duplex DNA (*τ* = 84 ns).^[^ ^89^ ^]^ More recently, a mononuclear ruthenium(II) complex coordinated to a **PhenDC3** derivative was shown to have distinct phosphorescence lifetimes for ssDNA, dsDNA, and G4s, with the latter being the longest.^[^ ^90^ ^]^ However, this phenomenon was not taken advantage of for cellular studies.

**Figure 9:**
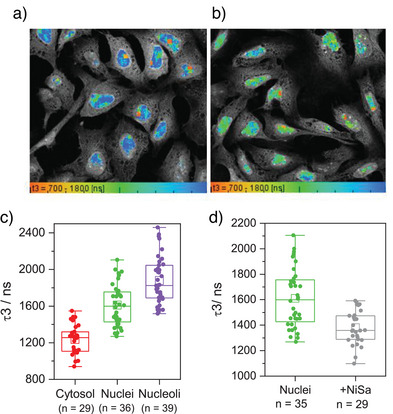
Detecting G4s with phosphorescence lifetime imaging microscopy (PLIM) with P3‐Pip.^[^ ^93^ ^]^ a), b) typical PLIM images of fixed U2OS cells stained with **P3‐Pip**, in the absence a) and the presence b) of 5 µM of the strong G4 binder **Ni‐Salphen**, the color scale is from 700 ns (red) to 1800 ns (blue), the long lifetimes observed in the nuclei are consistent with G4 binding *in cellulo*; c) PLIM data for **P3‐Pip** in fixed U2OS cells recorded from segmented nucleoli, whole nuclei and cytosol; the highest lifetime is seen in nucleoli is consistent with the highest proportion of G4 stained in this organelle and d) PLIM data for **P3‐Pip** in fixed U2OS cells in the presence of **Ni‐Salphen** (strong G4 binder, resulting in dye displacement from cellular G4s, leading to a lower lifetime); p < 0.05 for all condition pairs. Data reproduced with permission from Berrones Reyes et al. Platinum(II)‐based optical probes for imaging quadruplex DNA structures via phosphorescence lifetime imaging microscopy. *Angewandte Chemie International Edition*,^[^ ^93^ ^]^ Copyright Wiley 2023.

A series of cyclometallated platinum(II) complexes were reported to have distinctly longer phosphorescence lifetimes upon binding to G4 DNA as compared to other topologies.^[^ ^93^ ^]^ One of these complexes, **P3‐Pip** (Figure 8), showed intense nuclear staining in U2OS cells, with particularly bright emission in the nucleoli (Figure 9). Interestingly, the segmented PLIM images of the cell showed significantly longer phosphorescence lifetime in the nucleoli, followed by the lifetime in whole nuclei and much shorter lifetimes in the cytosol (Figure 9c), indicating the relative abundance of G4s stained in these organelles: nucleoli > whole nuclei > cytosol. To confirm that the long phosphorescence lifetimes observed in the nucleoli and nuclei of cells corresponded to the probe detecting G4s, **Ni‐Salphen** (a strong G4 binder that competes with the probe – see the FLIDA assay in Figure 6) was added to the cells. As expected, a reduction in the phosphorescence lifetime was observed (Figure 9d) which was consistent with the displacement of the platinum(II) probe from G4 DNA. While the mechanism of the lifetime contrast of phosphorescent probes upon binding to G4s is not yet known, this approach offers an attractive complementary technique for G4 detection, in addition to fluorescence staining. We note that alongside significant advantages offered by PLIM microscopy, there are limitations, such as weaker signals from phosphorescent probes, due to a spin‐forbidden nature of the emission, and limited availability of PLIM instrumentation, compared to FLIM. Given the signal originates from the triplet state of these probes, there is also a possibility of oxygen quenching of the probe's emission, and potential production of cytotoxic singlet oxygen, via an energy transfer, Type II, process from the triplet state.^[^ ^48^ ^]^

## 4 Ratiometric Emission Probes

**Figure 10:**
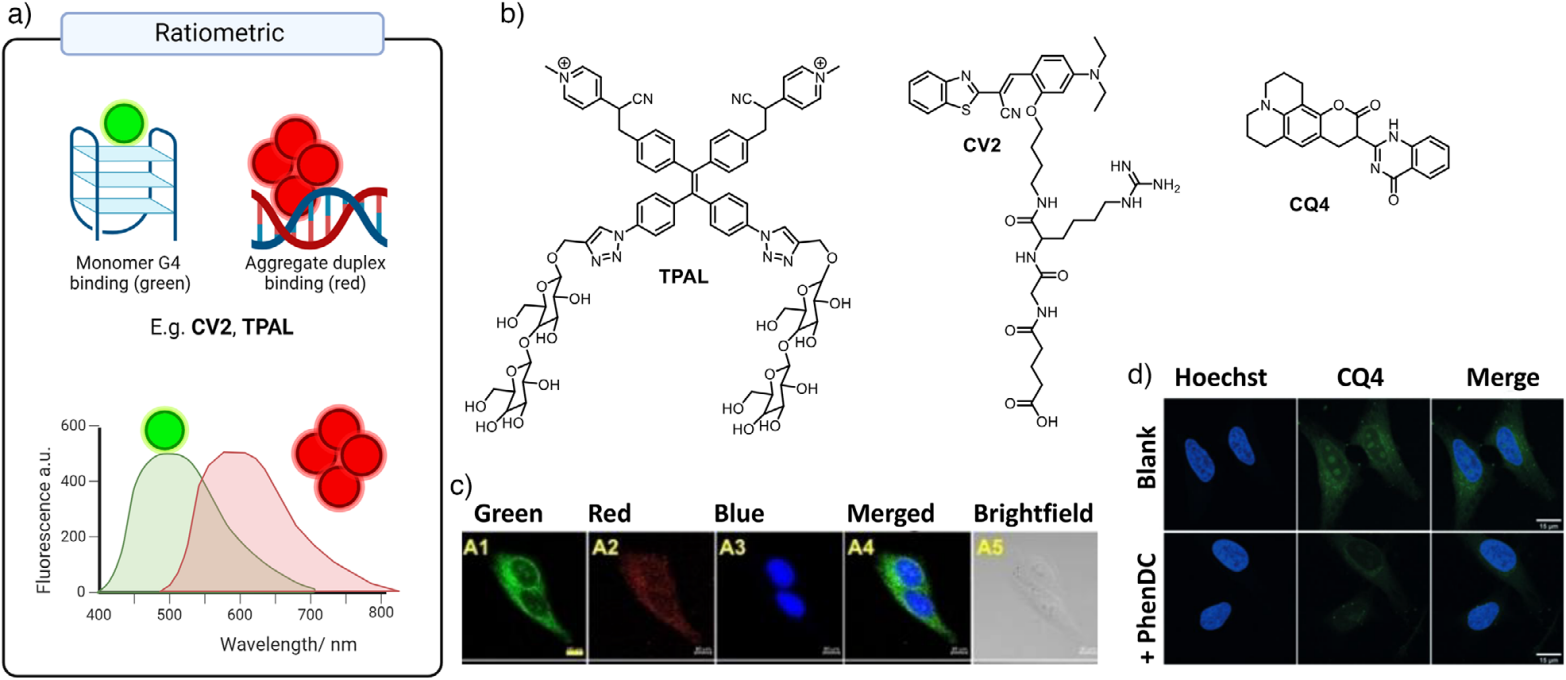
Working principles of ratiometric‐based G4 probes. a) The cartoon depiction of the spectral shift seen upon (dis)aggregation, where a monomeric form of the dye is favored by G4 binding, created with BioRender; b) the molecular structures of disaggregation‐induced emission based probes **TPAL**, **CV2,** and **CQ4**; c) confocal fluorescence images of fixed HeLa cells stained with **CV2** in green channel (monomer, G4), red channel (aggregate), blue (Hoechst, nuclei staining), and a merged image. Data reproduced with permission from M. Deiana et al. A Light‐up logic platform for selective recognition of parallel G‐quadruplex structures via disaggregation‐induced emission, *Angewandte Chemie International Edition*, Copyright Wiley 2020. d) Confocal fluorescence images of fixed HeLa cells stained with **CQ4** before (top) and after (bottom) the addition of competitive G4 binder **PhenDC3**. **CQ4** is detected in the green channel, Hoechst nuclear staining shows cell nuclei. Data reproduced with permission from A. Pandith et al., Self‐assembled peptidyl aggregates for the fluorogenic recognition of mitochondrial DNA G‐Quadruplexes, *Angewandte Chemie International Edition*, Copyright Wiley 2023.

An alternative approach to lifetime‐based detection of G4s, which allows problems of concentration‐dependent signal to be overcome, is to develop probes that have a unique spectral shift when bound to G4s. For instance, if G4 binding results in a large shift in emission wavelength of a probe, G4 interactions can be accurately detected by the appearance of a distinct peak in the spectrum, even if the probe interacts with many other structures that do not cause any spectral shifts (Figure 10). Such spectral‐based distinction was achieved by designing probes that exhibit disaggregation‐induced emission.^[^ ^94^, ^95^, ^96^ ^]^ In this case, small molecules self‐assemble into aggregates when free in solution exhibiting low and red‐shifted fluorescence. However, when interacting with G4s, the molecules disaggregate, which induces an increase in fluorescence intensity and, critically, a shift in the emission wavelength. An example of this type of probe is **CV2**, containing the excimer‐forming dye CV (Figure 10), which yields nanoaggregates with a red emission, but disaggregates in the presence of G4 DNA, returning to the bright green‐emitting form.^[^ ^94^ ^]^ Within cells, the ratio of the emission associated with the monomer (at 538 nm), relative to the emission of the aggregate (at 642 nm), could thus be used to monitor G4 formation in mitochondria, since no nuclear localization of the probe was observed (Figure 10c). The authors confirmed the reduction in the number of G4s detected upon addition of strong competitive ligands **PDS** and **PhenDC3**, and DNase. They further demonstrated the utility of their probe by showing that the removal of the G4 helicase PIF1 reduces the intensity of the emission associated with the monomer (corresponding to G4 binding), but not the emission of the aggregate (corresponding to non‐G4 interactions). Another optical probe based on disaggregation induced emission is **TPAL** (Figure 9b) whose disaggregation upon G4 binding results in a significant blue‐shift in emission.^[^ ^95^ ^]^ The authors confirmed that the cellular signal of the probe overlapped with a mitochondrial staining probe and was affected by DNase, but not RNase. The authors proceeded to use **TPAL** to monitor G4 foci dynamics in live HepG2 cells over time. Additionally, a coumarin‐quinazoline based dye **CQ4** that selectively lights‐up parallel G4 DNA structures via the disassembly of its supramolecular state was used for imaging G4s in the nuclei of fixed cells,^[^ ^96^ ^]^ including its displacement with **PhenDC3** (Figure 9d).

The di‐ruthenium complex shown in Figure 7, was one of the first switch‐on G4 DNA probes to be reported.^[^ ^89^ ^]^ The compound is practically nonemissive in aqueous solution, but upon interaction with DNA (both duplex and G4) it emits between 670 and 700 nm. Interestingly, in vitro studies showed that upon binding to antiparallel G4 from the human telomere sequence, the maximum in the emission of this probe is blue shifted (ca. 50 nm). In a recent study, the same authors separated two enantiomers of this compound and showed that the ΛΛ‐enantiomer is taken up by cells noticeably more rapidly than the ΔΔ‐form.^[^ ^97^ ^]^ More importantly, microscopy studies showed that although both isomers display a switch‐on of their emission, only the ΛΛ‐enantiomer displays the distinctive, blue‐shifted component associated with G4 DNA binding. Using MCF7 and L5178‐R cells, the probe was used to calculate the ratio of G4 versus duplex DNA over time. This showed that there is a decrease in the G4 content as cells undergo multiple division cycles, which would be consistent with the expected shortening of the telomeres.

An alternative method for achieving ratiometric monitoring of G4s is to conjugate a G4 probe to a reporter fluorophore whose fluorescence remains constant, irrespective of G4 or duplex binding.^[^ ^98^ ^]^ Thus, a red‐emitting small‐molecule fluorescent probe **DI** was conjugated to a quantum dot with green fluorescence.^[^ ^98^ ^]^ When free in solution or interacting with duplex DNA, the fluorescence of **DI** was low. However, binding of the probe to G4s dramatically increased its emission and resulted in a fluorescence color change in solution detectable by eye. This emission wavelength change was also used to detect G4s in cells via microscopy. As the green emission was not influenced by G4 binding, it could be used to normalize for probe concentration within cells, thus providing an additional concentration‐independent method of G4 visualization.

In a different take on ratiometric approach, the authors structurally modulated the green fluorescent protein (GFP)‐like chromophore by integrating the environmentally‐sensitive scaffold of GFP with coumarin 6H, obtaining a G4‐responsive and self‐calibrating dual‐emission probe, **NHCouI**.^[^ ^99^ ^]^ The red emission signal of **NHCouI** could specifically respond to parallel G4s, while its green emission signal was used as a concentration gauge. The authors studied the changing level of G4s in cells upon apoptosis and ferroptosis induction.

## 5 Single Molecule‐Based Probes

**Figure 11:**
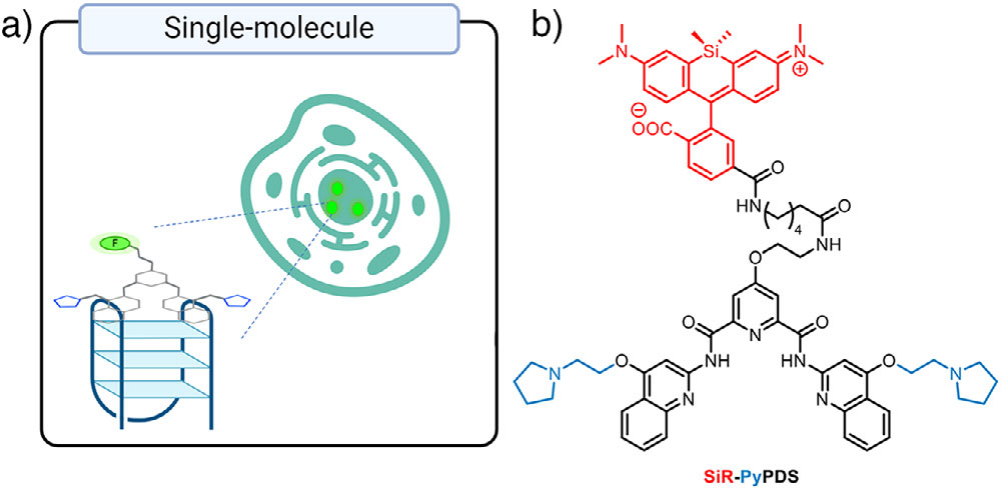
Working principles of single‐molecule‐based G4 probe. a) Cartoon depiction of the single molecule binding events detected probe in the cellular nuclei; b) the molecular structures of **SiRPy‐PDS**, in which the PyPDS unit strongly binds to G4s.^[^ ^100^ ^]^

One of the greatest limitations of intensity‐based switch‐on probes is the requirement for hyper‐selective G4 binding. However, nonselective interactions leading to undesirable non‐G4 staining can also be limited by greatly reducing the probe concentration used for imaging, for instance, by using highly sensitive G4 probes in single‐molecule detection mode. An example of a probe that has been used for this approach is **SiR‐PyPDS** (Figure 11).^[^ ^100^ ^]^ Typically, probe concentrations in the micromolar range are required for G4 imaging in cells which, as discussed above, may artificially induce G4 formation and lead to a large amount of background nonG4 signal. However, in single‐molecule imaging, only a fraction of all G4s that are folded in cells are imaged at a given time point, with compilation of images across time being used to provide a more global view of G4 formation. This fractional approach to G4 imaging requires a much lower concentration of the probe to be used, in the nanomolar rather than micromolar range. To demonstrate the G4 selectivity of **SiRPy‐PDS** within cells, the authors showed a significant reduction in binding events after displacement of the probe with G4 ligands (**PDS** and **PhenDC3**).^[^ ^100^ ^]^ The authors demonstrated that G4 formation in live cells is dependent on the stage of the cell cycle and could be disrupted by chemical inhibition of transcription and replication. Future development of single‐molecule approaches may therefore provide novel ways of dynamically imaging G4s in live cells without risk of small‐molecule induction of G4 formation.

## 6 Conclusions and Outlook

G‐quadruplexes (G4s) are considered to be one of the most abundant and biologically important noncanonical secondary structures of DNA and RNA. Despite a significant interest in their cellular roles, historically, their quantitative detection in live cells has been challenging, due to limited tools available. Significant milestones in G4 detection were achieved using strong‐binding antibodies such as BG4,^[^ ^9^ ^]^ however, their use in live cells is not possible. Large families of intensity‐based fluorescent probes have been designed, developed and utilized in in vitro experiments and in cellular imaging,^[^ ^41^, ^42^, ^45^, ^46^ ^]^ including “clickable” (such as PDS‐Alexa Fluor 594)^[^ ^61^ ^]^ and organelle‐targeted probes to visualize G4s in different cellular locations (e.g., mitochondria, cytoplasm, or nucleus).^[^ ^56^, ^57^, ^58^, ^59^, ^60^, ^61^ ^]^ However, there are concerns over false positive cellular signals that may be generated upon interactions of nontargeted “switch on” probes with nonG4 nucleic acids that are present in cellular environment in large excess.

This perspective reviewed recent advances in optical probes that utilize small‐molecule emitters that attempt false‐positive‐background‐free detection of changing G4 levels in live cells, without ambiguity imposed by excess duplex nucleic acids present in the cellular environment. As with any fluorescent probe, rigorous cellular testing of (photo)toxicity is essential prior to using the new molecules for elucidation of complex biological functions. Although some of the probes reviewed here, such as **P3‐Pip** and **TOR‐G4** were found to be toxic and could only be used in fixed cells,^[^ ^77^, ^93^ ^]^ other probes such as **DAOTA‐M2**,^[^ ^80^ ^]^ **ThT**,^[^ ^81^ ^]^ **SiR‐PyPDS**,^[^ ^100^ ^]^ and the di‐Ru complex shown in Figure 8,^[^ ^89^, ^90^ ^]^ could be successfully used to image G4s in live cells. This is in contrast to immunostaining which requires cell fixation.

The community access to the instrumentation and expertise required for these measurements should also be considered. Wide availability of confocal microscopy with multiwavelength detection means that the instrumentation required for ratiometric‐based G4 imaging is accessible to most chemistry and biology labs and can often be performed in fast dynamic mode. However, ratiometric probes are often complex synthetic fluorophores that have limited cell uptake and/or undesired localization or toxicity.

The diverse range of fluorescence lifetime‐based emitters has been developed and used in cells for G4 imaging, with many dyes suitable for live cell imaging, with no toxic effects displayed at the concentrations used. These new probes have facilitated novel and robust insights into G4 biology – from the role of various helicases unwinding G4s,^[^ ^78^ ^]^ to roles of G4 in cancer, and in mitochondrial functions.^[^ ^95^ ^]^ However, while there is an increasing number of these probes generating significant interest, including commercially available fluorophores,^[^ ^81^ ^]^ it has to be mentioned that these methods have not yet addressed many pressing demands in G4 cellular detection, such as fast dynamic imaging of G4s in physiological contexts or imaging G4s at specific genomic loci. FLIM microscopy requires a certain level of technical expertise, although this instrumentation is widely available in many microscopy facilities. The preferred mode of time‐resolved detection is often time‐correlated single photon counting (TCSPC) that allows high precision in lifetime determination yet slows down the acquisition due to low photon flux required and the need to collect sufficient photons for fitting of the time‐resolved decays in every image pixel accurately. Significant recent advances in camera‐based FLIM detection hold high promise for future possibilities of fast dynamic detection of lifetimes with good spatial resolution.^[^ ^101^, ^102^ ^]^ Additionally, super‐resolution FLIM imaging is feasible, utilizing STED FLIM,^[^ ^103^, ^104^ ^]^ although bright and photostable fluorophores are required for this approach to become practical. Although PLIM‐based detection offers several potential advantages over FLIM, this equipment is not currently widely available, and phosphorescent probes reported are not sufficiently bright to allow dynamic biological imaging.

Future development of single‐molecule approaches, similar to the one described in Figure 11, or even based on lifetime or ratiometric signal detection, may provide novel ways of dynamically imaging G4s in live cells at extremely low concentrations, without running the risk of artificially inducing G4 formation. However, these approaches will require the design of G4 specific probes of significantly increased brightness.

## Conflict of Interests

The authors declare no conflict of interest.

## Data Availability

Data sharing is not applicable to this article as no new data were created or analyzed in this study.
